# Baseline Prevalence of Trachoma in 13 Local Government Areas of Borno State, Nigeria

**DOI:** 10.1080/09286586.2022.2053550

**Published:** 2022-12-05

**Authors:** Mohammed Dantani Adamu, Aliyu Mohammed Jabo, Philomena Orji, Yaobi Zhang, Sunday Isiyaku, Nicholas Olobio, Nasiru Muhammad, Lawi Mshelia Auta, Rebecca Willis, Ana Bakhtiari, Cristina Jimenez, Anthony W. Solomon, Emma M. Harding-Esch, Caleb D. Mpyet

**Affiliations:** a Department of Ophthalmology, Usmanu Danfodiyo University, Sokoto, Nigeria; b Helen Keller International, Nigeria Country office, Abuja, Nigeria; c Helen Keller International, Regional Office for Africa, Dakar, Senegal; d Sightsavers Nigeria Country Office, Kaduna, Nigeria; e Federal Ministry of Health, Abuja, Nigeria; f Ministry of Health, Maiduguri, Nigeria; g International Trachoma Initiative, Task Force for Global Health, Decatur, Georgia, USA; h Sightsavers, Haywards Heath, UK; i Department of Control of Neglected Tropical Diseases, World Health Organization, Geneva, Switzerland; j Clinical Research Department, London School of Hygiene & Tropical Medicine, London, UK; k London Centre for Neglected Tropical Disease Research, London, UK; l Department of Ophthalmology, College of Health Sciences, University of Jos, Jos, Nigeria

**Keywords:** Nigeria, Borno, trachoma, trichiasis, prevalence, Tropical Data, WASH

## Abstract

**Purpose:**

We set out to determine the baseline prevalence of trachoma in 13 Local Government Areas (LGAs) of Borno State, Nigeria.

**Methods:**

A population-based cross-sectional survey was conducted in each of 13 LGAs from 2017 to 2019, with the support of Tropical Data (TD). World Health Organization (WHO)-recommended protocols were used. With a probability-proportional-to-size systematic sampling method, 25 villages were selected per LGA in 2017 and 30 villages per LGA in 2019; in each village, 25 households were enrolled for 2017 surveys, while 30 were enrolled for 2019 surveys. All present, consenting residents aged ≥1 year were examined by TD-certified graders for trachomatous inflammation—follicular (TF) and trachomatous trichiasis (TT) using the WHO simplified grading scheme. Additionally, we collected data on household-level access to water, sanitation and hygiene (WASH) facilities.

**Results:**

One LGA (Magumeri) had TF prevalence in 1–9-year-olds ≥10%; two other LGAs (Monguno and Kaga) had TF prevalence between 5.0% and 9.9%. The prevalence of TT unknown to the health system was ≥0.2% in six LGAs. The proportion of households with access to improved water sources ranged from 30% (Kwaya Kusar) to 95% (Monguno); household-level access to improved latrines was lowest in Shani (7%) and highest in Maiduguri (95%).

**Conclusion:**

Active TT case finding and strengthening of TT surgical services are needed in six LGAs. Mass drug administration (MDA) of antibiotics is needed in three LGAs to reduce the prevalence of active trachoma to below elimination thresholds. The trachoma elimination programme should engage WASH agencies to augment access to improved WASH facilities.

## Introduction

Trachoma is a chronic eye disease caused by *Chlamydia trachomatis*.^1^ With repeated infections – as occur over many years in people living in endemic populations – *C. trachomatis* can provoke conjunctival scarring and eventual in-turning of the eyelid. This may drive corneal scarring and blindness from the continuous rubbing of eyelashes on the cornea. Trachoma accounts for 4% of all blindness in Nigeria; within Nigeria, the north-eastern part of the country is thought to have the highest disease burden.^2,3^

The World Health Organization (WHO) recommends the SAFE strategy (**S**urgery for trichiasis; **A**ntibiotics to clear infection; **F**acial cleanliness and **E**nvironmental improvement to reduce transmission) to eliminate trachoma as a public health problem.^4^ Implementation of this strategy has been associated with reductions in trachoma prevalence in several countries, including Nigeria.^5–8^ WHO recommends that SAFE be applied at district level^4^ (which, for trachoma elimination purposes, is equivalent to the Local Government Area [LGA] in Nigeria; LGA populations range from 50,000 to 200,000 persons). The prevalence of trachomatous inflammation—follicular (TF) in children aged 1–9 years guides the need for A, F, and E, and the prevalence of trachomatous trichiasis (TT) unknown to the health system in adults aged ≥15 years guides the need for S. (TT “unknown to the health system” excludes TT that has already been operated on, which has a date set for surgery or for which management has been refused.)

Borno State is in the north-eastern Zone of Nigeria and shares boundaries with Adamawa, Gombe, and Yobe States of Nigeria, the Far North Region of Cameroon and the Diffa Region of the Republic of the Niger. In each of these adjacent administrative areas except Adamawa, trachoma has been documented to be a public health problem;^9–12^ in Adamawa, mapping of trachoma was undertaken in 21 LGAs in 2017–2019, revealing some TT but no LGAs with a TF prevalence at or above the 5% elimination threshold.^13^ Borno State has no established eye care programme, so no structured service has been provided for patients with trachoma and no recent local service data generated. It has also been facing security challenges for more than a decade,^14^ which have delayed field investigation.

We sought to generate LGA-level baseline data on trachoma in all 27 LGAs of Borno State. During the period of fieldwork, only 13 LGAs were accessible with safety. The remaining 14 LGAs were therefore omitted from this survey series; because of the ongoing insurgency in the state, it is unknown when surveys of these LGAs will be possible.^15^ The primary objective of this work was to estimate the LGA-level prevalence of TF in children aged 1–9 years. The secondary objectives were to estimate the LGA-level prevalence of TT unknown to the health system in individuals aged ≥15 years and to assess household-level access to WASH facilities in each LGA. WASH data assist with planning for the F and E components of SAFE. Joint monitoring of WASH and neglected tropical diseases also helps to detect inequalities, direct investments, and track progress.^16^

## Materials and methods

We conducted population-based cross-sectional surveys according to WHO design recommendations.^17,18^ Fieldwork took place between February 2017 (5 LGAs) and March 2019 (8 LGAs).

### Sample size calculation

The single-population-proportion-for-precision formula was used to determine the number of 1–9-year-old children to be enumerated to estimate an expected TF prevalence of 10% with an absolute precision of 3%. The sample size (n) was calculated as: n = DEFF × p(1-p)/(2 × d/((1.96 × 2)^2^)) × [non-response inflation factor]. We used a non-response rate inflation factor of 1.2. The design effect for cluster surveys was assumed to be 2.65,^18^ resulting in 1225 children to enumerate.

### Study design and sampling strategy

We used a two-stage sampling strategy to select the survey population. The first-stage clusters were villages. Since each survey team can reliably survey 25–30 households in 1 day, and there is a mean of 2.0 children aged 1–9 years in each rural household in northern Nigeria,^19^ for the 2017 surveys, 25 villages (1225/(25 × 2) = 24.5) were selected from a sampling frame, which consisted of a list of all villages in the LGA. However, for the 2019 surveys, to increase recruitment, 30 villages were selected per LGA. Villages were selected using systematic sampling with a probability-proportional-to-village-size methodology. Selection of households within selected villages was undertaken using the compact segment sampling method. Villages were divided into segments that contained approximately the same number of households (25 for 2017 surveys and 30 for 2019 surveys) and one segment was randomly selected by drawing lots. All households in this segment were visited. Within each household, all residents aged ≥1 year who had resided in the household for at least 6 months at the time of survey, including those absent at the time of visit, were identified by the household head and enumerated.

### Field team training

Tropical Data (TD, www.tropicaldata.org) protocols were followed in pre-survey field team training and certification. Version 2 of the TD training system was used for these surveys.^20^ All graders were ophthalmic nurses who had participated in a TD grader qualifying workshop and passed both the slide-based and live patient inter-grader agreement tests with kappa statistics for TF of ≥0.70. All recorders passed tests for fidelity of data recording.

### Individual-level data

Gender and age in years at last birthday were recorded for all household members. Those present at the time of the survey and willing to participate were examined for TT, TF, and trachomatous inflammation—intense (TI) by graders using 2.5× magnifying loupes and daylight illumination. Persons with TT were further examined for the presence of trachomatous scarring of the tarsal conjunctiva^7^ and asked whether they had previously been offered surgery or any other management for TT. In the 2019 surveys, follicle size guides^21^ were used to help graders consistently make a distinction between follicular inflammation that did or did not meet the definition of TF.^22^ For the purposes of this paper, we defined TT as the presence of trichiasis in either the upper or lower eyelid, with or without trachomatous conjunctival scarring. The teams made one return visit to households where children had been absent on first visit.

### Household-level data

GPS coordinates for each selected household were recorded. Data on WASH access were collected from the household head or their proxy. Household sanitation and handwashing facilities (if present) were then inspected to capture details of their type. Water sources and sanitation facilities were categorised as improved or unimproved, as per the WHO/UNICEF Joint Monitoring Program for Water Supply and Sanitation definitions used for monitoring progress towards the Sustainable Development Goals (SDGs).^18,23^ A household was defined as a household head together with all individuals normally resident in the compound and eating from the same pot.

### Data collection and management

All data were collected, stored, and managed using the TD system. Data were captured electronically using an Open Data Kit (ODK) smartphone application and sent to a secure Cloud-based server, then checked and cleaned by a dedicated data manager.^18^

### Ethics

Protocols were approved by the Ethics Committees of the Ministry of Health of Borno State and the National Health Research Ethics Committee of Nigeria (NHREC/01/01/2007). Approval for TD to support these surveys was given by the Ethics Committee of the London School of Hygiene & Tropical Medicine (16105). After field teams explained the examination protocol to each adult in a language that the person understood, informed verbal consent for enrolment and examination was obtained. Verbal consent was considered appropriate in this setting as adult literacy rates are low. Heads of households gave consent for the participation of minors, while adults gave consent for their own participation. Consent (or its refusal) was formally documented by the TD data recorder. Individuals with active trachoma (those found to have TF and/or TI) were given two tubes of 1% tetracycline eye ointment and instructed on its use. Persons with TT were referred for eyelid surgery at the nearest facility to the participant’s usual residence. Examiners cleaned their hands with an alcohol-based skin cleaning agent after examination of each participant.

Protecting our field staff was also an important ethical consideration. A security plan was devised in conjunction with security experts. Field security assessments were conducted ahead of the mapping and regularly revised during each survey. Survey teams were given specific security training and provided with satellite phones (Thuraya, United Arab Emirates) for use in case of emergency.

### Data analysis

Data were analysed in R (R Foundation for Statistical Computing, Vienna, Austria).^24^ Village-level TF proportions were adjusted for age in 1-year age bands. LGA-level TF prevalence estimates were calculated as the mean of adjusted village-level proportions. Village-level TT proportions were adjusted for gender and age in 5-year age bands. LGA-level TT prevalence estimates were calculated as the mean of the adjusted village-level proportions. Confidence intervals (CIs) were calculated by taking the 2.5th and 97.5th centiles of the distribution of prevalence after resampling the village means over 10,000 iterations.^18^

Surveys included the full package of TD quality assurance and quality control measures adopted from the Global Trachoma Mapping Project.^15^

## Results

Our field teams examined a total of 46,165 persons (Table 1). In the 1–9-year age group, 19,369 children were examined from 19,680 enumerated children, representing a response rate of 98.4% (Table 2). Of 23,675 ≥15-year-olds enumerated, 22,119 were examined, of whom 13,017 (58.8%) were females, representing a response rate of 93.4% (Table 3).

**Table 1.** Age and gender distribution of survey participants examined for trachoma, Borno State, Nigeria, February 2017–March 2019.

Age (years)	Females	%	Males	%	Total	% of Total
1–10	10,310	22	10,615	23	20,925	45
11–20	4175	9	2954	6	7129	15
21–30	4223	9	1766	4	5989	13
31–40	2815	6	1945	4	4760	10
41–50	1599	3	1623	4	3222	7
51–60	900	2	1136	2	2036	4
61–70	537	1	740	2	1277	3
71–80	282	1	338	1	620	1
≥81	85	0.2	122	0.3	207	0.5
Total	24,926	54	21,239	46	46,165	100

**Table 2.** Local Government Area (LGA)-level prevalence of trachomatous inflammation—follicular (TF), Borno State, Nigeria, February 2017–March 2019.

LGA		1–9-year-olds		Age-adjusted TF prevalence in 1–9-year-olds	
	Number of Clusters surveyed	Enumerated	Examined	(%) (95% CI)	
**2017 surveys**					
Bayo	25	1179	1166	2.8	(1.6–4.5)
Biu	25	975	947	0.8	(0.3–1.4)
Hawul	25	1005	992	1.0	(0.5–1.8)
Kwaya Kusar	25	1077	1073	2.6	(1.4–4.0)
Shani	25	1044	1032	1.5	(0.7–2.2)
**2019 surveys**					
Askira/Uba	30	1801	1792	1.1	(0.6–1.6)
Chibok	30	1824	1813	1.4	(0.7–2.2)
Damboa	30	1724	1716	3.4	(1.7–5.6)
Jere	30	1962	1916	3.2	(1.7–5.2)
Kaga	30	1900	1888	5.9	(4.3–7.9)
Magumeri	30	1849	1844	12.2	(8.5–16.2)
Maiduguri	30	1761	1658	4.6	(2.4–6.8)
Monguno	30	1579	1532	8.4	(5.5–11.9)

**Table 3.** Prevalence of trachomatous trichiasis (TT) unknown to the health system and TT backlog in 13 Local Government Areas (LGAs), Borno State, Nigeria, February 2017–March 2019.

LGA	Estimated total population of persons aged ≥15 years^a^	≥15 years		Age- & gender-adjusted TT prevalence in ≥15-year-olds		
				Prevalence^b^ (%)	(95% CI)	Estimated TT backlog^c^
		Enumerated					Examined
**2017 surveys**						
Bayo	44,284	1358	1302	0.29	(0.10–0.56)	128
Biu	98,426	1549	1489	0.19	(0.03–0.43)	0
Hawul	67,610	1454	1378	0.04	(0.00–0.09)	0
Kwaya Kusar	31,754	1479	1418	0.09	(0.02–0.19)	0
Shani	56,554	1389	1306	0.15	(0.04–0.28)	0
**2019 surveys**						
Askira/Uba	80,255	2284	2123	0.11	(0.01–0.27)	0
Chibok	37,146	2320	2179	0.06	(0.00–0.14)	0
Damboa	130,592	1867	1784	0.56	(0.29–0.99)	731
Jere	117,100	2189	1917	0.64	(0.13–1.50)	749
Kaga	50,398	1906	1799	0.35	(0.13–0.65)	176
Magumeri	78,544	1831	1757	0.4	(0.19–0.67)	314
Maiduguri	302,409	2155	1890	0.18	(0.03–0.40)	0
Monguno	61,507	1894	1777	0.79	(0.20–1.82)	486
**Total**	**1,156,579**					**2584**

CI, confidence interval.

^a^Based on data from the 2006 census.^25^

^b^Prevalences of TT unknown to the health system are displayed to two decimal places in order to provide clarity on whether or not the best estimate of prevalence was above or below the elimination threshold of 0.2% in adults ≥15 years.

^c^TT backlog was calculated as per methods previously described.^26^

### Prevalence of trachoma

**Figure 1.**
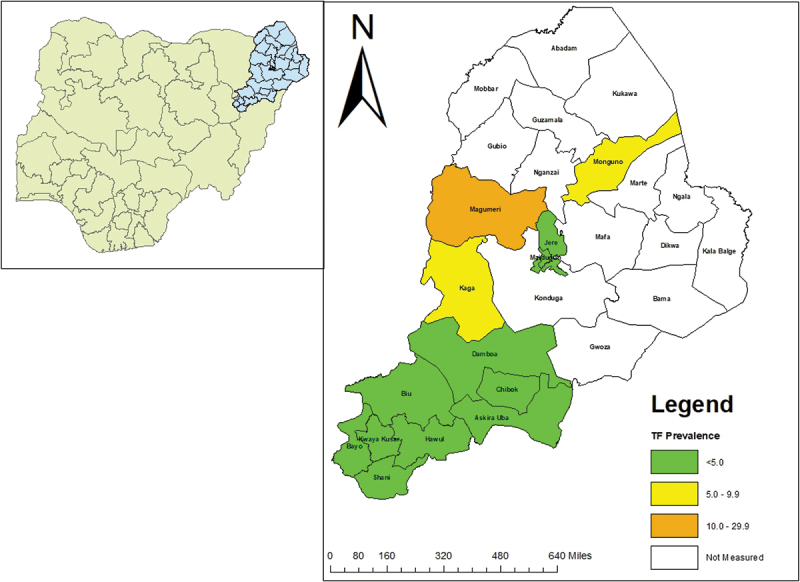
Prevalence of trachomatous inflammation—follicular (TF) in 1–9-year-old children, by Local Government Area, Borno State, Nigeria, February 2017–March 2019. The boundaries and names shown and the designations used on this map do not imply the expression of any opinion whatsoever on the part of the authors, or the institutions with which they are affiliated, concerning the legal status of any country, territory, city or area or of its authorities, or concerning the delimitation of its frontiers or boundaries.

**Figure 2.**
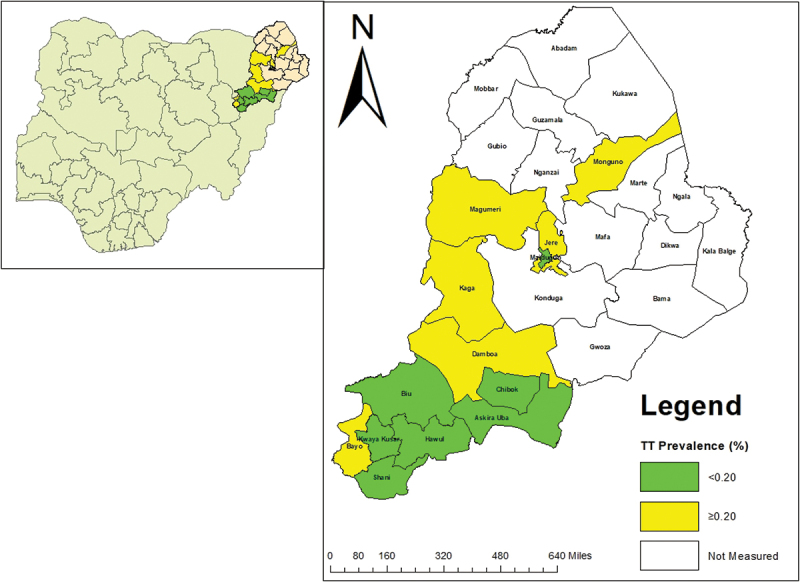
Prevalence of trachomatous trichiasis (TT) unknown to the health system in persons aged ≥15 years, by Local Government Area, Borno State, Nigeria, February 2017–March 2019. The boundaries and names shown, and the designations used on this map do not imply the expression of any opinion whatsoever on the part of the authors, or the institutions with which they are affiliated, concerning the legal status of any country, territory, city or area or of its authorities, or concerning the delimitation of its frontiers or boundaries.

The prevalence of TF in children aged 1–9 years ranged from 0.8% in Biu to 12.2% in Magumeri; Magumeri was the only LGA with a TF prevalence of ≥10%. Two LGAs (Kaga and Monguno) had TF prevalence estimates between 5.0% and 9.9% (Table 2 and Figure 1). The prevalence of TT unknown to the health system in persons aged ≥15 years was <0.2% in seven LGAs (range 0.04–0.19%) and ≥0.2% in six LGAs (range 0.29–0.79%) (Table 3 and Figure 2). The TT backlog in these 13 LGAs, which in 2006 had an estimated combined population of 2,065,320,^25^ was 2584 (Table 3).

### Water and sanitation coverage

The proportion of households with access to improved drinking water sources was lowest in Kwaya Kusar (30%) and highest in Monguno (95%). Only three (23%) of the 13 LGAs had ≥80% of households with access to an improved drinking water source. Reported drinking water access within 30 minutes of the household ranged from 49% in Kwaya Kusar to 79% in Bayo. Similarly, across the state, there was poor access to improved latrines (latrines that hygienically separate human faeces from human contact), with only one LGA having ≥80% household-level improved latrine access (Table 4). Household-level access to improved latrines was lowest in Shani (7%) and highest in Maiduguri (95%; Table 4).

**Table 4.** Household access to water, sanitation, and hygiene facilities in 13 local government areas (LGAs), Borno State, Nigeria, February 2017–March 2019.

LGA	Households surveyed	Households with an improved drinking water source N (%)	Households with drinking water source within a 30-minute return journey N (%)	Households with an improved latrine N (%)
Bayo	616	245 (40)	485 (79)	91 (15)
Biu	620	442 (71)	478 (77)	400 (65)
Hawul	625	250 (40)	388 (62)	208 (33)
Kwaya Kusar	623	186 (30)	305 (49)	180 (29)
Shani	625	226 (36)	469 (75)	44 (7)
Askira/Uba	896	389 (43)	634 (71)	613 (68)
Chibok	902	448 (50)	601 (67)	625 (69)
Damboa	897	643 (72)	637 (71)	643 (72)
Jere	898	598 (67)	570 (63)	706 (79)
Kaga	898	771 (86)	513 (57)	536 (60)
Magumeri	895	803 (90)	452 (51)	442 (49)
Maiduguri	896	497 (55)	649 (72)	848 (95)
Monguno	901	855 (95)	473 (52)	512 (57)

## Discussion

The prevalence of TF was above the threshold for intervention for elimination as a public health problem in three of the 13 Borno State LGAs we surveyed: Magumeri, Monguno, and Kaga. These LGAs qualify for antibiotic mass drug administration (MDA) for trachoma elimination purposes as well as initiatives focused on facial cleanliness and environmental improvement, though the evidence base for the A component of SAFE currently outweighs that for F and E.^27–29^ Current WHO recommendations suggest that prior to undertaking impact surveys, Magumeri LGA should receive 3 years of A, F, and E, while Monguno and Kaga should each receive 1 year of A, F, and E.^30^ Additionally, since each of those LGAs, plus Bayo, Damboa, and Jere, had prevalence estimates for TT unknown to the health system above the elimination threshold, six LGAs require programmes of active TT case finding and community-based eyelid surgery to clear the estimated TT backlog of approximately 2,500 people, all of whom are at risk of imminent sight loss. In the other LGAs, incident and post-operative TT management should be managed through routine eye health-care services.^31^ However, Borno State currently has no existing eye care programme and no formally trained TT surgeons. Individuals with TT must therefore consult one of the relatively few in-state ophthalmologists to access TT management, a situation that has undoubtedly contributed to the above-threshold TT prevalences. To achieve and maintain elimination of trachoma here, it will be necessary to train and equip TT surgeons in Borno, then provide an appropriate structure for facilitating their work. Surgical training should follow guidelines established by WHO.^32^ Regular supportive supervision is needed to maximise service productivity and quality.^33^

Delivering these activities represents a somewhat herculean task considering the profound security challenge (the ongoing insurgency) facing the state and its neighbours. However, we learned a lot from conducting these surveys in areas with security difficulties, and it may be helpful to translate that experience to the planning of interventions. Some examples of practices which could be adopted include staying in contact with local authorities; plying safe routes to reach affected communities; training people living within or close to affected communities to undertake surgeries and fieldwork; offering to engage in dialogue on trachoma elimination with leaders of combatant groups, maintaining political neutrality in any discussion; and, if necessary, avoiding the use of GPS or electronic data capture.^34–37^ Use of local hire cars instead of marked project vehicles and transporting patients to safer areas for service delivery may also help to minimise exposure of health workers to excess risk.

In all surveyed LGAs, with the exception of the state capital, Maiduguri, less than 80% of surveyed households had access to improved latrines. There were no LGAs in which >80% of households had a water source within 30 minutes round trip of the house, and there were only three LGAs in which >80% of households had an improved washing water source. This highlights the need for provision of these amenities across all surveyed LGAs. This needs to be done to help prevent possible recrudescence of active trachoma. Though prospective data are scarce, cross-sectional data suggest that populations with high-coverage access to sanitation are less likely to have active trachoma.^38,39^ Governments, sanitation agencies, and other partners should prioritise WASH provision across the state as soon as security conditions allow. This should complement ongoing efforts to achieving SDG 6: “Ensuring availability and sustainable management of water and sanitation for all”.^40^

Given the generally poor access to clean water sources and improved sanitation facilities across these LGAs, it is somewhat surprising that 10 of the 13 LGAs had TF prevalence estimates <5%. A possible explanation may be a reduction in *C. trachomatis* transmission intensity due to curtailing of social interactions, occasioned by the insurgency, which at the time of these surveys had been ongoing for 10 years.

Our survey methodology followed WHO recommendations for survey design parameters^41^ despite the security challenges we faced in the field. However, there are some unavoidable weaknesses. Though the 2019 survey employed 30 clusters in line with the recent WHO recommendations for precision on TT prevalence,^174342,43^ the 2017 surveys were powered to detect TF prevalence and not TT prevalence. Reliance on self-reporting of access to water and use of sanitation may introduce biases, although direct observation was employed where possible to confirm responses. Protocol modifications adopted during this challenging survey series should be mentioned here: In the second phase of the survey (2019) involving eight LGAs, the number of selected clusters was increased from 25 to 30 and the number of households also increased from 25 to 30 to increase the sample size, as the number of children examined in the 2017 LGAs was low. In addition, our field itinerary was repeatedly revised to adapt to the rapidly changing security situation; this may have affected recruitment success if changes in our rendezvous dates in particular villages decreased enthusiasm for participation. Finally, our definition of TT does not allow for analysis of the results in line with the new definition of TT as agreed at the 4th Global Scientific Meeting on Trachoma^44,45^ – this meeting recommended that TT be defined as at least one eyelash from the upper eyelid or evidence of recent epilation of inturned eyelashes from the upper eyelid, thereby excluding trichiasis affecting only the lower eyelid. The inclusion of lower lid trichiasis in our surveys may have inflated our TT prevalence estimates.

## Conclusion

Through careful planning combined with flexibility, it was possible to conduct these surveys despite security challenges. At least six of Borno State’s LGAs require a TT surgery programme and at least three require implementation of the A, F and E components of the SAFE strategy for trachoma elimination purposes. Improvements in access to WASH facilities are required across the board to achieve the SDG water and sanitation targets.

## References

[1] Taylor HR, Burton MJ, Haddad D, West S, Wright H. Trachoma. *Lancet*. 2014;384(9960):2142–2152. doi:10.1016/S0140-6736(13)62182-0. PMID: 25043452. 25043452

[2] Kyari F, Gudlavalleti MV, Sivsubramaniam S, et al. Prevalence of blindness and visual impairment in Nigeria: the national blindness and visual impairment survey. *Invest Ophthalmol Vis Sci*. 2009;50(5):2033–2039. doi:10.1167/iovs.08-3133. 19117917

[3] Smith JL, Sivasubramaniam S, Rabiu MM, Kyari F, Solomon AW, Gilbert C. Multilevel analysis of trachomatous trichiasis and corneal opacity in Nigeria: the role of environmental and climatic risk factors on the distribution of disease. *PLoS Negl Trop Dis*. 2015;9(7):e0003826. doi:10.1371/journal.pntd.0003826. PMID: 26222549. 26222549 PMC4519340

[4] Francis V, Turner V. *Achieving Community Support for Trachoma Control (WHO/PBL/93.36)*. Geneva: World Health Organization; 1993.

[5] Ngondi J, Onsarigo A, Matthews F, et al. Effect of 3 years of SAFE (surgery, antibiotics, facial cleanliness, and environmental change) strategy for trachoma control in southern Sudan: a cross-sectional study. *Lancet*. 2006;368(9535):589–595. doi:10.1016/S0140-6736(06)69202-7. 16905023

[6] Hammou J, El Ajaroumi H, Hasbi H, Nakhlaoui A, Hmadna A, El Maaroufi A. In Morocco, the elimination of trachoma as a public health problem becomes a reality. *Lancet Glob Health*. 2017;5(3):e250–e1. doi:10.1016/S2214-109X(17)30023-2. 28089329

[7] Ferriman A. Blinding trachoma almost eliminated from Morocco. *BMJ*. 2001;323(7326):1387. doi:10.1136/bmj.323.7326.1387b. 11744558 PMC1121857

[8] Mpyet C, Muhammad N, Adamu MD, et al. Impact survey results after SAFE strategy implementation in fifteen Local Government Areas of Kebbi, Sokoto and Zamfara States, Nigeria. *Ophthalmic Epidemiol*. 2018;25(sup1):103–114. doi:10.1080/09286586.2018.1481984. PMID: 30806537. 30806537 PMC6444276

[9] Mpyet C, Ogoshi C, Goyol M. Prevalence of trachoma in Yobe State, north-eastern Nigeria. *Ophthalmic Epidemiol*. 2008;15(5):303–307. doi:10.1080/09286580802237633. 18850466

[10] Mpyet C, Muhammad N, Adamu MD, et al. Trachoma mapping in Gombe State, Nigeria: results of 11 local government area surveys. *Ophthalmic Epidemiol*. 2016;23(6):406–411. doi:10.1080/09286586.2016.1230633. 27726459 PMC6839962

[11] Noatina BN, Kagmeni G, Mengouo MN, et al. Prevalence of trachoma in the Far North region of Cameroon: results of a survey in 27 Health Districts. *PLoS Negl Trop Dis*. 2013;7(5):e2240. doi:10.1371/journal.pntd.0002240. 23717703 PMC3662655

[12] Cromwell EA, Amza A, Kadri B, et al. Trachoma prevalence in Niger: results of 31 district-level surveys. *Trans R Soc Trop Med Hyg*. 2014;108(1):42–48. doi:10.1093/trstmh/trt101. 24281748

[13] Adamu MD, Jabo AM, Orji P, et al. Baseline Local Government Area-Level Prevalence of Trachoma in Adamawa State, North East: results of 21 population-based prevalence surveys. *Ophthalmic Epidemiol*. 2021;1–9. doi:10.1080/09286586.2021.2013899.

[14] Medecins Sans Frontieres. Lake Chad crisis: over 10 million people heavily dependent on aid for survival. Federal identification number: UID CH-660.1.555.004-1. Accessed 13 Dec 2021. https://www.msf.org/lake-chad-crisis-depth.

[15] Solomon AW, Willis R, Pavluck AL, et al. Quality assurance and quality control in the global trachoma mapping project. *Am J Trop Med Hyg*. 2018;99(4):858–863. doi:10.4269/ajtmh.18-0082. PMID: 30039782. 30039782 PMC6159583

[16] Boisson S, Engels D, Gordon BA, et al. Water, sanitation and hygiene for accelerating and sustaining progress on neglected tropical diseases: a new Global Strategy 2015-20. *Int Health*. 2016;8(Suppl 1):i19–i21. doi:10.1093/inthealth/ihv073. PMID: 26940305. 26940305 PMC5580794

[17] World Health Organization Strategic and Technical Advisory Group on Neglected Tropical Diseases. Design parameters for population-based trachoma prevalence surveys (WHO/HTM/NTD/PCT/2018.07). Geneva: World Health Organization; 2018.

[18] Solomon AW, Pavluck AL, Courtright P, et al. The Global Trachoma Mapping Project: methodology of a 34-country population-based study. *Ophthalmic Epidemiol*. 2015;22(3):214–225. doi:10.3109/09286586.2015.1037401. 26158580 PMC4687001

[19] National Population Commission. *Housing Characteristics and Amenities Tables – Priority Tables (LGA)*. Vol. II. Abuja, Nigeria: National Population Commission: National Population Commission. 2006.

[20] Courtright P, MacArthur C, Macleod C, et al. *Tropical Data: Training System for Trachoma Prevalence Surveys*. London: International Coalition for Trachoma Control; 2016.

[21] Solomon AW, Le Mesurier RT, Williams WJ. A diagnostic instrument to help field graders evaluate active trachoma. *Ophthalmic Epidemiol*. 2018;1–4. doi:10.1080/09286586.2018.1500616. PMC6850902 30067432

[22] Thylefors B, Dawson CR, Jones BR, West SK, Taylor HR. A simple system for the assessment of trachoma and its complications. *Bull World Health Organ*. 1987;65:477. 3500800 PMC2491032

[23] *Core Questions on Drinking Water and Sanitation for Household Surveys*. Geneva: World Health Organization; 2006.

[24] R Core Team. 2020. R: a language and environment for statistical computing. R Foundation for Statistical Computing, Vienna, Austria. https://www.R–project.org. Accessed Dec 7, 2021.

[25] National Population Commision. *National and State Population and Housing Tables: Priority Table 1&43.Abuja*. National Population Commission: National Population Commission; 2006.

[26] Solomon AW, Bella ALF, Negussu N, Willis R, Taylor HR. How much trachomatous trichiasis is there? A guide to calculating district–level estimates. *Community Eye Health*. 2019;31:S5. 31086446 PMC6390516

[27] Evans JR, Solomon AW, Kumar R, et al. Antibiotics for trachoma. *Cochrane Database Syst Rev*. 2019;9:CD001860. DOI:10.1002/14651858.CD001860.pub4. PMID: 31554017. PMC6760986 31554017

[28] Ejere HO, Alhassan MB, Rabiu M. Face washing promotion for preventing active trachoma. *Cochrane Database Syst Rev*. 2015;2:CD003659. doi:10.1002/14651858.CD003659.pub4. PMID: 25697765. PMC4441394 25697765

[29] Rabiu MM, Alhassan MB, Ejere HO, Evans JR. Environmental sanitary interventions for preventing active trachoma. *Cochrane Database Syst Rev*. 2012;2:CD004003. doi:10.1002/14651858.CD004003.pub4. PMID: 22336798. PMC4422499 22336798

[30] World Health Organization. Technical consultation on trachoma surveillance: meeting report. September 11− 12, 2014. Task Force for Global Health. Decatur, USA. Geneva: World Health Organization; 2015

[31] The International Coalition for Trachoma Control (ICTC). Transition planning for trichiasis management services. https://www.trachomacoalition.org/sites/default/files/content/resources. Accessed March 2019.

[32] Merbs S, Resnikoff S, Kello AB, Mariotti S, Greene G. *West SK.Trichiasis Surgery for Trachoma*. Geneva: World Health Organization; 2013.

[33] Lewallen S, Mahande M, Tharaney M, Katala S, Courtright P. Surgery for trachomatous trichiasis: findings from a survey of trichiasis surgeons in Tanzania. *Br J Ophthalmol*. 2007;91(2):143–145. doi:10.1136/bjo.2006.102368. 16973662 PMC1857600

[34] Elshafie BE, Osman KH, Macleod C, et al. The epidemiology of trachoma in Darfur States and Khartoum State, Sudan: results of 32 population-based prevalence surveys. *Ophthalmic Epidemiol*. 2016;23(6):381–391. doi:10.1080/09286586.2016.1243718. 27841721 PMC5297557

[35] Ko R, Macleod C, Pahau D, et al. Population-based trachoma mapping in six evaluation units of Papua New Guinea. *Ophthalmic Epidemiol*. 2016;23(sup1):22–31. doi:10.1080/09286586.2016.1235715. PMID: 27893297. 27893297 PMC5706965

[36] Thabit AA, Al-Khatib T, Hail WHM, et al. Prevalence of trachoma in Yemen: results of population-based prevalence surveys of 42 evaluation units in nine governorates. *Ophthalmic Epidemiol*. 2018;25(sup1):62–69. doi:10.1080/09286586.2018.1441426. 30806535 PMC6444195

[37] Yaya G. Lessons learned in the implementation of programmes to eliminate trachoma within conflict zones. *Trans R Soc Trop Med Hyg*. 2021; In press. 10.1093/trstmh/trac061 PMC9623735 36027048

[38] Garn JV, Boisson S, Willis R, et al. Sanitation and water supply coverage thresholds associated with active trachoma: modeling cross-sectional data from 13 countries. *PLoS Negl Trop Dis*. 2018;12(1):e0006110. doi:10.1371/journal.pntd.0006110. PMID: 29357365. 29357365 PMC5800679

[39] Oswald WE, Stewart AE, Kramer MR, et al. Active trachoma and community use of sanitation, Ethiopia. *Bull World Health Organ*. 2017;95(4):250–260. doi:10.2471/BLT.16.177758. 28479620 PMC5407250

[40] Gupta J, Vegelin C. Sustainable development goals and inclusive development. *Int Environ Agreements Polit Law Econ*. 2016;16(3):433–448. doi:10.1007/s10784-016-9323-z.

[41] Engels D. The global trachoma mapping project: a catalyst for progress against neglected tropical diseases. *Ophthalmic Epidemiol*. 2016;23(sup1):1. doi:10.1080/09286586.2016.1257139. PMC5706979 28030282

[42] World Health Organization. *Design and Validation of a Trachomatous Trichiasis-only Survey*. Geneva: World Health Organization. (WHO/HTM/NTD/PCT/2017.08). Licence: CC BY-NC-SA 3.0 IGO; 2017.

[43] Courtright P, MacArthur C, Macleod C, et al. *Tropical Data: Training System for Trachoma Prevalence Surveys (Version 2)*. London: International Coalition for Trachoma Control; 2016.

[44] Solomon AW, Kello AB, Bangert M, et al. The simplified trachoma grading system, amended. *Bull World Health Organ*. 2020;98(10):698–705. doi:10.2471/BLT.19.248708. 33177759 PMC7652564

[45] World Health Organization. Report of the 4th Global Scientific Meeting on Trachoma, Geneva. Geneva: World Health Organization. Accessed 13 Dec 2021. https://apps.who.int/iris/bitstream/handle/10665/325121/WHO-CDS-NTD-PCT-2019.03-eng.pdf?ua=1.

